# Sequenced genomes and rapidly emerging technologies pave the way for conifer evolutionary developmental biology

**DOI:** 10.3389/fpls.2015.00970

**Published:** 2015-11-03

**Authors:** Daniel Uddenberg, Shirin Akhter, Prashanth Ramachandran, Jens F. Sundström, Annelie Carlsbecker

**Affiliations:** ^1^Physiological Botany, Department of Organismal Biology and Linnean Centre for Plant Biology, Uppsala BioCenter, Uppsala University, Uppsala, Sweden; ^2^Department of Plant Biology and Linnean Centre for Plant Biology, Uppsala BioCenter, Swedish University of Agricultural Sciences, Uppsala, Sweden

**Keywords:** gymnosperms, plant developmental biology, plant evo-devo, next-generation sequencing, plant transformation

## Abstract

Conifers, Ginkgo, cycads and gnetophytes comprise the four groups of extant gymnosperms holding a unique position of sharing common ancestry with the angiosperms. Comparative studies of gymnosperms and angiosperms are the key to a better understanding of ancient seed plant morphologies, how they have shifted over evolution to shape modern day species, and how the genes governing these morphologies have evolved. However, conifers and other gymnosperms have been notoriously difficult to study due to their long generation times, inaccessibility to genetic experimentation and unavailable genome sequences. Now, with three draft genomes from spruces and pines, rapid advances in next generation sequencing methods for genome wide expression analyses, and enhanced methods for genetic transformation, we are much better equipped to address a number of key evolutionary questions relating to seed plant evolution. In this mini-review we highlight recent progress in conifer developmental biology relevant to evo-devo questions. We discuss how genome sequence data and novel techniques might allow us to explore genetic variation and naturally occurring conifer mutants, approaches to reduce long generation times to allow for genetic studies in conifers, and other potential upcoming research avenues utilizing current and emergent techniques. Results from developmental studies of conifers and other gymnosperms in comparison to those in angiosperms will provide information to trace core molecular developmental control tool kits of ancestral seed plants, but foremost they will greatly improve our understanding of the biology of conifers and other gymnosperms in their own right.

## Can we Establish a Conifer Model Species for Developmental Studies?

**FIGURE 1:**
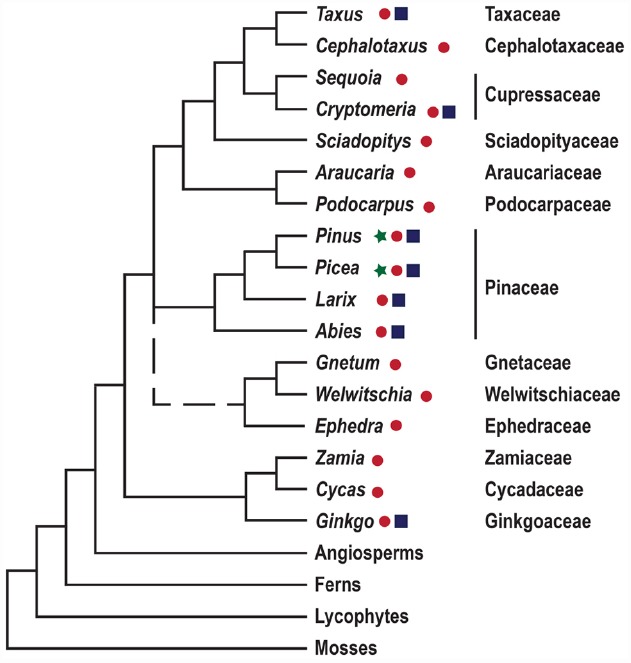
**A simplified depiction of land plant phylogeny.** The tree is based on the comprehensive studies of seed plant phylogeny by Wickett et al. (2014) and Ruhfel et al. (2014). Gymnosperm genera for which genome and transcriptome sequence data are available are highlighted by ⋆ and ● respectively. Genera in which transformation protocols have been established are indicated by ■. Gnetophytes are represented by a dashed line since their position in the phylogenetic tree remains unresolved.

Conifers are of great ecological and economic importance; they dominate the forests of the northern hemisphere, and comprise two thirds of extant gymnosperms (Wang and Ran, 2014). Seed plants, constituting gymnosperms and angiosperms, evolved 300–350 million years ago, and their appearance is defined by the evolution of the ovule. The subsequent evolution of seed plants resulted in the elaboration of reproductive organ morphologies, including the innovation of the flower and carpel in the angiosperm lineage, but is also associated with, e.g., variations in embryo morphologies and water and assimilate conducting tissues (Taylor et al., 2009). To understand the evolution of novel morphologies we need to put these traits into a phylogenetic context. However, the deep branches of the seed plant phylogeny have been notoriously difficult to resolve, and the relative position of conifers, gnetophytes, cycads and *Ginkgo*, remains a focus for research (Figure 1; Wang and Ran, 2014). Over the last decades evolutionary developmental biology (evo-devo), has surfaced as an approach adding to traditional systematic efforts. Evo-devo studies rely on comparative analyses of the genetic mechanisms underlying the development of certain morphological traits, as exemplified by the evolution of reproductive structures in seed plants, see Mathews and Kramer (2012).

Currently, we have extensive knowledge on developmental genetic mechanisms mainly from a handful of angiosperm model species, primarily *Arabidopsis thaliana* (*Arabidopsis*). This is because ideal models typically are small, self-fertile, have short generation times, small genomes sizes and are amenable to genetic transformation (The Arabidopsis Genome Initiative, 2000). Gymnosperms, on the other hand, comprise long-lived perennial woody species with large population sizes, high degree of heterozygosis, and huge genomes sizes, and therefore lack model organism characteristics. The current phylogenetically narrow focus on selected pines and spruces has instead largely been the result of geo-economical decisions. However, recent advances in molecular techniques have laid the foundation for a knowledge leap toward revealing the underlying genetic mechanisms controlling important traits also in species that lack the typical characteristics of model species.

## Next Generation Sequences and Genetic Transformation—Conifer Developmental Biology Studies Made Possible

The development of next-generation sequencing (NGS) techniques has surfaced as one of the most important technological breakthrough in current biology (Wang et al., 2009), making genomes and transcriptomes available from both model and non-model species. For non-model plants, such resources include draft sequences of the 20–30 Gigabase genomes from *Picea abies*, *P. glauca*, and *Pinus taeda* (Figure 1; Birol et al., 2013; Nystedt et al., 2013; Neale et al., 2014). These initiatives revealed that although the genomes are huge, largely owing to accumulation of long-terminal repeat transposable elements, the numbers of protein-coding sequences are similar to angiosperms. The draft conifer genome sequences and accompanying transcriptome data can be found in dedicated, constantly updated databases, aiming to help researchers navigate this vast amount of data^1^^,^^2^. These data and corresponding databases will serve as an essential foundation for future studies.

The development and improvement of single-cell “omics” will probably drive the next advancement in genetic and transcriptomic research (Junker and van Oudenaarden, 2014), and, moreover, methods to retain positional information of the cell (*in situ* “omics”) promise to shed light also on the spatial regulation (Crosetto et al., 2015). Although technology development in this area still is in its early days and in large remains to be adapted to plants, this second avenue of NGS techniques will open up for more fine-tuned systems biology approaches, allowing computational and mathematical modeling of, e.g., transcription factor and signaling pathways.

Functional studies are crucial to test hypotheses of biochemical activity and forward genetic screens have therefore been imperative in identifying novel key developmental regulators in angiosperms. Previously, this relied on mapping using recombinant mapping populations, but NGS now allows sequencing of entire genomes, thus dramatically speeding up cloning of the causal mutation in model systems, and potentially making forward genetic screens possible in non-model systems (Schneeberger, 2014). Techniques that allow for NGS of particular genomic regions or transcribed loci, i.e., exome sequencing, may also help to overcome problems of genome complexity and SNP discovery in non-model systems (Neves et al., 2014).

**FIGURE 2:**
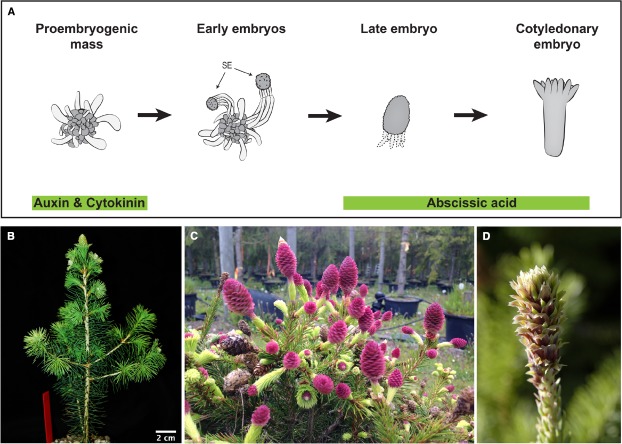
**Emerging tools for conifer functional studies: Embryogenic cultures and the rapid cycling ***P. abies acrocona*** mutant. (A)** Schematic representation of somatic embryogenesis in *P. abies*: Embryogenic cultures are routinely established from zygotic embryo explants via the addition of auxin and cytokinin to culture media. Proliferating cultures consist of proembryogenic masses (PEMs), a mixture of densely cytoplasmic meristematic cells and large vacuolated cells. Withdrawal of auxin and cytokinin stimulates differentiation of early somatic embryos (SE) from PEMs. Early somatic embryos consist of apically located meristematic cells of the embryo proper, embryonal tube cells in the central region and a tier of terminally differentiated suspensor cells. Further embryo development and maturation requires the addition of abscisic acid. During late embryogeny apical meristems are formed and the suspensor cells undergo programmed cell death. Mature cotyledonary embryos are formed after 4–8 weeks on maturation medium. Image after Filonova et al. (2000). **(B)** Early cone setting in an inbred *acrocona* plant after three growth cycles (1 year) (Uddenberg et al., 2013). **(C)** Massive cone production in an older inbred *acrocona* plant. **(D)** *Acrocona* vegetative branch transitioning into female reproductive state. Needles gradually converts into bracts and ovuliferous scales appear in their axils. This transition is accompanied by the onset of a number of putative key reproductive developmental regulators (Carlsbecker et al., 2013).

Assessments of gene function require the generation of mutants or transgenes with altered gene activity, caused by knock-out, knock-down, or over-expression of specific loci. In species with long life cycles such as gymnosperms genetic transformation over seed generations is not possible. However, this can be circumvented by utilizing somatic embryogenesis, in which proliferating embryogenic tissue is transformed by direct DNA delivery or via bacteria-mediated horizontal gene transfer. This method has been employed to generate transgenic conifer tissues for many years (Figure 2A; Tang and Newton, 2003). Some conifer and gymnosperm species are more recalcitrant to genetic transformation; however, the efficacy of transformation has greatly improved, mainly by using hypervirulent *Agrobacterium* strains and improved protocols, now facilitating the generation of stably transformed plants from many conifer species (Levee et al., 1997; Wenck et al., 1999; Klimaszewska et al., 2001; Le et al., 2001; Alvarez and Ordás, 2013). The use of embryo explants during transformation, followed by selective tissue culture and plant regeneration, provide an alternative for recalcitrant species (Tang et al., 2014).

## Conifer Somatic Embryos Enable Functional Evolutionary Developmental Biology

Somatic embryogenesis is used in certain conifer species as a method for large-scale clonal propagation, facilitating long-term storage of germplasm, and as a tool in breeding programs. This technique also offers an efficient and versatile tool to study the morphology and underlying molecular regulation of conifer embryonal traits. Somatic embryo systems allow closer studies of the establishment of the plant basal body plan, including apical-basal specification, formation of the apical meristems and patterning of the dermal, ground and procambial tissues (Smertenko and Bozhkov, 2014). Studies in *Arabidopsis* have shown that these processes depend on distinct spatio-temporal action of certain transcription factors and local biosynthesis and polar transport of the plant hormone auxin (Ten Hove et al., 2015). Studies on the effect of chemical inhibition of polar auxin transport during somatic embryo development of *P. abies* show increased levels of endogenous auxin, decreased programmed cell death (PCD) activity and abnormal suspensor differentiation during early stages of embryogenesis. Later stages treated with the chemical display both basal and apical aberrations, including fused cotyledons and unorganized meristems (Larsson et al., 2007; Hakman et al., 2009), suggesting a conserved role for auxin in basic embryo formation in seed plants. Comparative studies of homologs to angiosperm key factors for embryo patterning and polarity such as *WUSCHEL-RELATED HOMEOBOX* (*WOX*) genes and class I *KNOTTED1-like homeobox* (*KNOX1*) genes, using transgenic conifer somatic embryos, suggest considerable conservation but also functional divergence (Belmonte et al., 2007; Zhu et al., 2014; Alvarez et al., 2015). Potentially, Less biased methods such as global gene expression analyses during both somatic and zygotic embryogenesis indicate a significant overlap in transcript profiles of developmental regulators between conifers and angiosperms, but also reveal many genes of unknown function active during embryogenesis, emphasizing the need for future comparative functional studies (Vestman et al., 2011; de Vega-Bartol et al., 2013).

Interestingly, the first plant metacaspase involved in PCD was originally discovered in *P. abies.* Functional studies suppressing the type II metacaspase, *mcII-Pa*, in somatic embryos of *P. abies* showed that it is an essential component of vacuolar cell death, required for normal development and degradation of suspensors during early embryogenesis (Suárez et al., 2004) and that it acts via an autophagy-related pathway (Minina et al., 2013). Further studies, initiated in *P. abies* have also demonstrated that plant PCD share common genetic components with PCD in animals and humans (Sundström et al., 2009). Hence, conifer somatic embryogenesis provides an excellent system, not only for comparative studies, but also to identify novel regulators of general developmental processes.

## Gymnosperm Reproductive Development Through a Genomic Lens: ABC or Only BC?

The evolution of the flower remains a major unresolved question in biology, since transition forms have not been reliably identified in the fossil record and extant gymnosperms are only distantly related to the angiosperms (Frohlich and Chase, 2007). While the angiosperms and gymnosperms are united by the feature of producing ovules, their reproductive organs are distinct: In contrast to the hermaphroditic angiosperm flower with the stamens and carpels surrounded by a sterile perianth of sepals and petals, the reproductive organs in gymnosperms are formed from separate meristems. Furthermore, the gymnosperm organs carrying the ovules have very distinct morphologies compared to the angiosperm carpel, preventing reliable inferences of organ homologies based on their morphology. However, despite morphological diversity, evo-devo-studies show that molecular mechanisms controlling the development of the reproductive organs of angiosperms and gymnosperms are at least partially conserved (Melzer et al., 2010; Mathews and Kramer, 2012).

The identities of the flower organs are based on conserved key regulatory transcription factors, and were summarized in the ABC-model: A-function specify sepals, A together with B specifying petals, B and C specify stamens, whereas C alone specifies the carpel (Coen and Meyerowitz, 1991). The ABC-model was based on studies in *Arabidopsis* and has at least in part, been shown valid for most angiosperms, although the A-function have been assigned to floral meristem identity rather than to sepal identity in some angiosperms (Litt and Irish, 2003). Support for conserved molecular mechanisms for reproductive organ identity determinations among the seed plants came with the identification of putative orthologs to B- and C-genes in several gymnosperm species, along with the finding that both the B- and C-homologs are active specifically in developing male cones (Mouradov et al., 1999; Sundström et al., 1999; Winter et al., 1999) whereas C-function homologs also are active during the formation of the ovule bearing organs of the female cones (Tandre et al., 1995, 1998; Rutledge et al., 1998), leading to the hypothesis that B and C together specifies male reproductive identity, and C alone female reproductive identity in all seed plants.

Most gymnosperm female cones have a compound architecture, with ovule-bearing structures subtended by bracts, and neither female nor male cones have structures with apparent homology to the sterile perianth (sepals and petals). In line with this, PCR-based methods, aimed at identifying a broad range of MADS-box genes, failed to identify gymnosperm genes orthologous to the A-type MADS-box genes (Shindo et al., 1999; Winter et al., 1999; Carlsbecker et al., 2013). For a long time, it was considered an established fact that gymnosperms lacked both perianth-like organs and associated regulatory genes. In the first analysis of the *P. abies* genome, however, Nystedt et al. (2013) observed a remarkable expansion of the MADS-box gene family. Among the staggering 249 Type II MADS-box genes in the *P. abies* genome at least one gene group in a clade including both angiosperm A-function and FLOWERING LOCUS C-genes (Gramzow et al., 2014), calling for a reexamination of a potential A-function in conifers.

## Teens for Decades—Can we Overcome the Long Generation Time of Gymnosperms to Facilitate Developmental Genetic Studies?

Gymnosperms are in general perennial trees, or shrubs, and most take decades until they enter the reproductive phase. Therefore, all functional evidence of any gene active in reproductive development in gymnosperms comes from testing their effect on the development of rapid cycling angiosperms (e.g., Tandre et al., 1998; Winter et al., 2002; Karlgren et al., 2011). Thus, there is a great need to better understand the molecular control of juvenile–adult and vegetative–reproductive transitions in gymnosperms, and if possible establish a more rapidly cycling model (Uddenberg et al., 2013). Currently, most knowledge of developmental transitions comes from the annual plant *Arabidopsis*, although studies of perennial angiosperm trees, in particular poplar, promise to add important knowledge for comparative analyses with gymnosperms (Böhlenius et al., 2006; Wang et al., 2011). Not surprisingly, transitions may be controlled by distinct mechanisms in annuals and perennials, and in angiosperms and gymnosperms. In angiosperms, key regulators of the transition from vegetative to reproductive phase are orthologs of *FLOWERING LOCUS* T (*FT*) from *Arabidopsis* (Wigge et al., 2005). Although conifers possess *FT* homologs, studies in Norway spruce indicated that they lack *FT* orthologs (Karlgren et al., 2011; Klintenäs et al., 2012), a notion confirmed with the sequencing of the spruce genome (Nystedt et al., 2013). Another conserved angiosperm key regulator acting upstream of the ABC-genes is *LEAFY* (*LFY;* Moyroud et al., 2010). While gymnosperms do have an apparent *LFY* ortholog they also have a paralogous gene, called *NEEDLY* (Mellerowicz et al., 1998; Mouradov et al., 1998; Vazquez-Lobo et al., 2007). Currently available data is not informative to reveal if these genes may confer similar functions as their angiosperm counterpart.

Much of what we know about reproductive development in angiosperms is based on functional analysis of individual genes using mutants, either in forward or reverse genetic approaches. Interestingly, several varieties of conifers with peculiar reproductive structures or other phenotypes are available in arboretums (Rudall et al., 2011), and natural variants are alternatives to classic forward genetic screens (Dosmann and Groover, 2012). A naturally occurring mutant of *P. abies*, called *acrocona*, produces cones frequently, even in years when surrounding trees rarely set cones (Figures 2B–D). Inbred crosses show that a quarter of the segregating siblings initiate cones extremely early, already during their second growth season (Figure 2B; Uddenberg et al., 2013), and a single locus of importance for the cone setting phenotype has been mapped to a specific chromosome (Achere et al., 2004). Hence, the segregation pattern suggests that the early cone setting phenotype is caused by a monogenic loci and further analyses of its phenotype that it is likely semidominant (Uddenberg et al., 2013). NGS of *acrocona* transcriptomes (RNA-seq) identified a candidate gene related to the angiosperm floral integrator *SUPPRESSOR OF THE OVEREXPRESSION OF CONSTANS 1 (SOC 1;* Lee and Lee, 2010*)* that may be involved in the early cone-setting phenotype (Uddenberg et al., 2013).

In addition to the early and frequent cone-setting, the *acrocona* mutant produces vegetative shoots transformed into reproductive cones, by initiation of ovuliferous scales in the axil of needles (Figures 2C,D). Detailed expression analysis using mRNA *in situ* hybridization have been used to study regulatory genes with putative functions in reproductive initiation, organ identity and pattern formation in wild type, male and female cones as well as in the *acrocona* transition shoots, as a means of testing of hypotheses of function by assessing gene expression correlation with the initiation and formation of ectopic female structures (Carlsbecker et al., 2013). Hence, already now, without knowing the nature of the causal mutation, *acrocona* allows further studies of putative reproductive development genes. These may include MADS-box genes hypothesized to control phase transitions (Carlsbecker et al., 2003, 2004), or the newly identified putative A-class homolog (Gramzow et al., 2014). Like their angiosperm homologs (Litt and Irish, 2003), these genes may initiate reproduction in *P. abies*, and their activity can be analyzed in the mutant background. NGS of dissected tissues in various developmental phases in wild types and mutants will allow detailing such studies further.

## Feeding Conifer Developmental Biology into Breeding Programs

In addition to the key evolutionary position occupied by the gymnosperms, a strong driving force to further our knowledge about their development, reflected in the two *Picea* and one *Pinus* species chosen for full genome sequences, is the economic importance of conifer wood. Wood is formed from the vascular meristem, the cambium. Although its activity is essential for all tree species, determining growth rate, wood formation and quality, it is among meristems the least understood. Most of our understanding of cambium activity and wood formation comes from studies of *Arabidopsis* and Poplar. These studies have revealed transcriptional and hormonal control mechanisms for cambium and wood formation as well as the biosynthesis pathways for cellulose, hemicellulose, and lignin (Lucas et al., 2013). Promisingly, comparative studies of genomes and transcriptomes have revealed a substantial conservation of regulatory mechanisms for cambium and wood formation between angiosperms and conifers (Li et al., 2010; Carvalho et al., 2013). Now, systems biology approaches will likely rapidly enhance our knowledge of conifer wood formation, beyond a mere comparison with more tractable angiosperm models. These approaches include co-expression analyses, transcription factor–promoter and protein–protein interaction analyses (Duval et al., 2014), in combination with assessments of transgenic seedlings with perturbed putative key regulators (Bomal et al., 2008). In addition, analyses of naturally occurring mutants, such as the cinnamyl alcohol dehydrogenase mutant defective in lignin formation (Ralph et al., 1997), will be important to connect wood properties and growth parameters. Knowledge gained could be used to generate computational models for vascular development, increase our understanding of specific features distinguishing angiosperm and gymnosperm secondary development and improve early stage identification of desirable traits important for breeding of economically important conifer species.

## Outlook

As costs for current sequencing methods decrease and third and fourth generation techniques such as nanopore sequencing are taken into general use (Feng et al., 2015), we can envision a more diversified sampling of sequenced organisms within the gymnosperm lineage, together with the assembly of high quality genomes. Better sequence information can be used to develop denser maps of short nucleotide polymorphisms (SNPs), enabling genome-wide marker-based selection and allowing more efficient breeding, as well as utilization of natural variation in studies of developmental control mechanisms. Emerging quality updates on reference genomes will also most likely facilitate the establishment of methods such as CRISPR/Cas9 (Belhaj et al., 2015), greatly increasing the possibility to generate single and multiple mutants. A continued development of efficient techniques to generate inducible genes, the development of strong fluorescent reporters coupled with better detection techniques will likely revolutionize functional studies of at least early stages of conifer development.

The long generation time of most gymnosperms makes any attempt to perform functional studies of adult characters or even simple breeding efforts a time-consuming endeavor. Once the causing mutation of the early cone-setting phenotype of the *acrocona* mutant is known, it will be a potentially powerful tool to generate rapid cycling lines not only in *P. abies*, but perhaps also in other transformable conifers and gymnosperms. This would enable functional studies of regulatory genes implicated in juvenile–adult transition, as well as reproductive initiations and reproduction organ specification. It may also allow the transfer of introduced traits from primary transformants to consecutive generations. Hence, new and emerging technologies promise a blooming future for conifer developmental biology, as well as for evo-devo studies in gymnosperms.

### Conflict of Interest Statement

The authors declare that the research was conducted in the absence of any commercial or financial relationships that could be construed as a potential conflict of interest.

## Footnotes

1 http://dendrome.ucdavis.edu/

2 http://congenie.org/
